# Identification of a partnership model between a university, for-profit, and not-for-profit organization to address health professions education and health inequality gaps through simulation-based education: A scoping review protocol

**DOI:** 10.1371/journal.pone.0288374

**Published:** 2023-07-10

**Authors:** Samyah Siraj, Beheshta Momand, Ginny Brunton, Adam Dubrowski

**Affiliations:** Faculty of Health Sciences, Ontario Tech University, Oshawa, Ontario, Canada; Access Alliance Multicultural Health and Community Services: Access Alliance, CANADA

## Abstract

**Introduction:**

Healthcare providers in rural and remote (R&R) areas of Canada do not have the same access to skills development and maintenance opportunities as those in urban areas. Simulation-based education (SBE) is an optimal technique to allow healthcare providers to develop and maintain skills. However, SBE is currently limited mainly to universities or hospital-based research laboratories in urban areas. The purpose of this scoping review is to identify a model, or components of a model, that outline how a university research laboratory can collaborate with a for profit and not-for-profit organization to facilitate the diffusion of SBE into R&R healthcare provider training.

**Methods and analysis:**

This scoping review will be guided by the methodological framework introduced by Arksey and O’Malley in 2005 and the Methodology for Joanna Briggs Institute Scoping Reviews. Ovid MEDLINE, PsycINFO, Scopus, Web of Science, and CINAHL will be searched for relevant articles published between 2000 and 2022, in addition to grey literature databases and manual reference list searches. Articles describing a partnership model or framework between academic institutions and non-profit organizations with a simulation or technology component will be included. Titles and abstracts will be screened, followed by a full-text screening of articles. Two reviewers will participate in the screening and data extraction process for quality assurance. Data will be extracted, charted, and summarized descriptively to report key findings on potential partnership models.

**Conclusion:**

This scoping review will provide an understanding on the extent of existing literature regarding the diffusion of simulators for healthcare provider training through a multi-institutional partnership. This scoping review will benefit R&R parts of Canada by identifying gaps in knowledge and determining a process to deliver simulators to train healthcare providers. Findings from this scoping review will be submitted for publication in a scientific journal.

## Introduction

The field of health professions education (HPE) recognizes that all healthcare providers should be competent and have access to optimal training resources to develop and maintain competencies [1]. Healthcare providers in rural and remote (R&R) areas of Canada, more specifically physicians and nurses, may not have the same access to skills development and maintenance opportunities as those in urban areas due to factors such as distance from urban centers and cost [2]. The inexperience and lack of training for healthcare providers is one factor that has impacted the quality of care received by Canadians in R&R settings [3]. One example is a lack of training on technical skills used to perform high-acuity low-occurrence (HALO) procedures. HALO procedures are rarely performed clinical procedures that are required to be performed urgently when needed [3]. Having limited training on how to perform HALO procedures can affect the health outcomes of a patient in emergency situations and in locations where accessing addition health care services is difficult. The College of Family Physicians of Canada (CFPC) highlights the need to develop contextual competencies of providers to practice in R&R clinical settings and recognizes the need to reform HPE due to the significance the level of education, in relation to training and competencies, has on adequately serving the health needs of the population [3]. To improve rural education programs, one of the objectives that the CFPC has identified is to develop tools to increase access to clinical training, with one such tool being simulation technology [3].

Simulation-based education (SBE) is defined as a replication of a real task (or patient encounter) for the purpose of training or assessment of quality improvement and is proven to be a key aspect of HPE [4]. SBE serves as an optimal technique to allow healthcare providers to develop and maintain skills and can be used for assessment and evaluation without endangering the safety of a patient [5]. The use of additive manufacturing allows for the improved availability of affordable simulators through a flexible, efficient, and sustainable production process that maintains high quality and low cost [6]. The most known form of additive manufacturing is three-dimensional (3D) printing. 3D printing technologies are a useful tool in medical training and healthcare due to its customizability to fit any context and the reduction of costs in production [7]. However, the development of simulators using additive manufacturing and 3D printing technologies is currently limited mainly to university or hospital-based research laboratories in urban areas of developed countries, and are rarely diffused to R&R settings, where they can create a greater impact [2, 8, 9]. Siraj et al. and Barth et al. report on the positive outcomes of using SBE and 3D printing technologies to train students and healthcare providers using simulators to learn and improve hands-on clinical skills [9, 10]. Developing simulators in a research laboratory using 3D printing technologies can help reduce costs and address gaps in HPE for R&R healthcare providers [10].

An example of a simulator developed in a university research laboratory include a 3D printed/silicone simulator to train on the intraosseous (IO) access skill, where a hole is drilled into the proximal tibia bone to deliver fluids and medication into the bone marrow [11]. The material cost to manufacture a simple IO simulator is $12.66 CAD and costs $53.89 CAD to manufacture an advanced IO simulator. When compared to a commercially available IO similar that retails for $414 USD, the simulator developed in the laboratory is produced at a fraction of the cost [11]. Another example is the development of a cost-effective cricothyroidotomy simulator for emergency medicine simulation training. Cricothyroidotomy is a procedure that allows for tracheal intubation in life-threatening situations [12]. The material cost of developing this anatomically accurate simulator using 3D printing technology is $3.63 CAD [12]. The cost of developing the simulator is covered through research and development funding whereby the research laboratory designs, prototypes and conducts research in the areas of validity, acceptability, feasibility and efficacy/effectiveness [11, 12]. Once the research and development process has concluded, the healthcare system requires these simulators to be manufactured to address their training needs, however, there is no mechanism or model to facilitate this process.

Public health systems research (PHSR) is a growing focus in Canada that looks at financing, delivery, and impact of public health services [13]. One of the top six priorities of PHSR in Ontario is partnerships and linkages. This priority highlights the importance of partnerships between various sectors (i.e. healthcare providers, educational institutions, community-based organization, government, etc.) to improve the performance of the public health system [13]. Establishing partnerships between different sectors, such as university research laboratories, for-profit organizations (FPOs), and not-for-profit organizations (NPOs), can improve capacity building of healthcare providers and knowledge exchange within that healthcare system. To date, there are no specific SBE-focused partnership models that address how 3D printed simulation technology can make its way from university research laboratories into the HPE system, especially in the R&R context. Establishing a partnership between relevant organizations can facilitate the process of delivering simulators that are developed in the lab to hospitals and educational institutions in R&R parts of Canada to train healthcare providers. A multi-institutional partnership can help overcome logistical challenges experienced by one sector, such as manufacturing the simulator, by pooling resources to provide simulators to R&R healthcare providers in a way that is beneficial to all partners [1, 14]. Making SBE available to communities in need can improve health outcomes through its link to healthcare provider training [15]. Therefore, the purpose of this scoping review is to understand the existing literature and identify if a model exists that outlines how a university research laboratory can collaborate with a FPO and NPO.

## Protocol design

The methodological framework introduced by Arksey and O’Malley [16] will be used to guide this scoping review by adhering to the five stages of the framework: 1) identifying the research question, 2) identifying relevant studies, 3) study selection, 4) charting the data, and 5) collating, summarizing and reporting the results. The scoping review will be conducted and reported in adherence with the Joanna Briggs Institute Manual for Evidence Synthesis’ scoping review chapter [17], and the Preferred Reporting Items for Systematic reviews and Meta-Analyses extension for Scoping Reviews (PRISMA-ScR) Checklist [18]. This study does not require ethics approval as the scoping review methodology involves reviewing and collecting data from publicly available materials.

### Stage 1: Identifying the research question

The purpose of this scoping review is to understand the existing literature and identify if a model exists that outlines how a university research laboratory can collaborate with a FPO and NPO. The research question developed based on this purpose and in consultation with the research team is: What are the existing partnership models, or components of a model, that outline how a university research laboratory can collaborate with a FPO and NPO to deliver technological solutions to the healthcare education sector? Preliminary searches suggest that there is no specific model that is focused on this type of partnership. Therefore, comparable models will be chosen that can provide information for the creation of a new model specific to the research question. To accomplish this, the specific objectives of the scoping review are 1) to identify partnership models that can be adapted to support ways a university research laboratory can collaborate with a FPO and NPO to deliver SBE solutions to healthcare providers in R&R settings, and 2) to identify strategies that can be used in a stage of the model in relation to a partnership.

### Stage 2: Identifying relevant studies

Five literature databases will be searched for this scoping review: Ovid MEDLINE, PsycINFO, Scopus, Web of Science, CINAHL. Concepts derived from the research question will be used to form the free-text codes for the search strategy on Ovid MEDLINE which will then be translated to the four remaining databases. Keywords such as “academic institution”, “partnership”, “not-for-profit”, “simulation”, and “healthcare” and synonym terms will be used. The free-text codes will be combined with database-specific controlled subject headings to form the search strategy on the five published literature databases. The free-text and controlled subject heading codes used for the search strategy on Ovid MEDLINE are shown in S1 Table. Limitations will be placed on the search engines for the time frame and the article language to ensure only relevant articles appear in the results.

Grey literature will be searched on the following databases to mitigate the possibility of limited results from published literature. The following grey literature databases will be searched: Grey Matters (CADTH), OpenGrey, and Google Scholar. In addition, the reference list of relevant articles will be manually searched to identify new articles that address the research question. The time frame for the search will be 2000 to 2022, as simulation officially became an area of scientific inquiry aiming to standardize its use in HPE after 2000 to reduce the number of medical errors occurring by healthcare providers [19]. The search strategy has been developed in consultation with a health science librarian as per PRESS guidelines [20].

### Stage 3: Study selection

The citation management software Endnote 20 will be used for initial screening to organize the selected articles and remove article duplicates. After refining the search query, the results will be imported to the EPPI-Reviewer software for screening and selection. Screening of the articles will follow a two-step screening process. The first step will be to screen titles and abstracts to determine the eligibility of each article. Publications will be excluded if the title or abstract does not meet the inclusion criteria. The second step will be a full-text screening of articles that have passed the first step and only relevant articles will be included in the review. Reporting of the study selection will be done using the PRISMA flow diagram. Study designs that will be accepted for the review are scoping and systematic reviews, surveys, case studies, systematic reviews, mixed methods studies, and commentaries/ program evaluations. Articles will be selected using specific criteria for inclusion and exclusion, to ensure the articles address the specific research question. The inclusion and exclusion criteria have been chosen to account for the relevance of the article to the context of the question and to find articles that could address or provide information on the proposed gap. The inclusion criteria include the need for the article to describe a partnership model or framework, involve both academic institutions and non-profit organizations, focus on post-secondary education, and have a simulation/technology component. The exclusion criteria include articles that focus on kindergarten to grade school (secondary education), editorial articles, conference abstracts, posters, or dissertations and articles not published in English. The eligibility criteria may change depending on the search results and relevance of the studies.

### Stage 4: Charting the data

The data will be extracted and charted using the EPPI-Reviewer software. The following key elements will be extracted from the articles: author details, country of origin, study objective/purpose, study design, technology developed, participant characteristics, sentences defining ’partnership model’ or ‘collaboration model’, and key findings related to the research question.

### Stage 5: Collating, summarizing and reporting the results

In accordance with the purpose of this scoping review, potential models that address the research question will be reported. If no model is identified, models that come close will be presented and characteristics of the model that can contribute to the development of a new partnership model will be summarized. Quantitative and qualitative data will be summarized descriptively in text and presented in tables and graphs where appropriate. Components of the models identified from selected articles that are relevant to the partnership process and addresses the research gap will be synthesized and grouped as themes. Strategies identified from the selected articles that can be applied in the different stages of a partnership process will be classified as subthemes under the themes created.

### Quality assurance

Endnote 20 is the citation management tool that will be used to organize articles after executing the search strategy and to create references for selected articles [21]. Following this, the EPPI-Reviewer software, a web-based software program used to manage and analyze data in literature reviews will be used to screen and select articles [22]. Two reviewers (SS & BM) will participate in the screening and data extraction process. Both reviewers will independently screen the title and abstract of a subset of the search results (10%) to assess the eligibility criteria and perform a quality check. Both reviewers will then meet to discuss their ratings and refine the eligibility criteria as needed. Disagreements will be resolved by a third reviewer where needed. Following this exercise, one reviewer (SS) will screen the remaining references with any questions checked by a second reviewer (BM). During the charting stage, data will be extracted from 100% of the studies by one reviewer (SS), and from two random samples of 5% each by a second reviewer (BM) to ensure charting quality.

## Dissemination

Results of this scoping review will provide an understanding on the extent of existing literature on diffusing simulators to R&R areas for healthcare provider training through a multi-institutional partnership. Additionally, the results will help identify the gaps in knowledge to establishing and sustaining a partnership that has a SBE focus. The results of this scoping review will be relevant and informative for various stakeholders: academic bodies, researchers, NPOs, simulation technologists, and healthcare providers, as a partnership addressing the identified research gap can be mutually beneficial to all stakeholders involved. In relation to dissemination, findings from this scoping review will be submitted for publication to a peer-reviewed journal.

## Strengths and limitations of the study

- This scoping review will be the first to examine literature to identify a partnership model that focuses on implementing SBE practices, especially in the Canadian R&R context.
- The identification of articles will be done through five published literature databases, three grey literature databases, and snowball references.
- Based on preliminary searches, there is no model that specifically addresses the research question. Therefore, comparable models will be chosen that can provide information for the creation of a new model specific to the research question.

## Supporting information

S1 Checklist PRISMA-P checklist. (DOCX)

S1 Table Draft search strategy for Ovid MEDLINE. (DOCX)
